# Synthesis of 6-Amino-5-cyano-1,4-disubstituted-2(1*H*)-Pyrimidinones via Copper-(I)-catalyzed Alkyne-azide ‘Click Chemistry’ and Their Reactivity

**DOI:** 10.3390/molecules15128841

**Published:** 2010-12-03

**Authors:** Ennaji Najahi, Jan Sudor, Fakher Chabchoub, Françoise Nepveu, Fethi Zribi, Romain Duval

**Affiliations:** 1 Laboratory of Applied Chemistry, Heterocycles, Fats and Polymers, Faculty of Science, University of Sfax, 3000 Sfax, Tunisia; E-Mails: najahimco@yahoo.fr (E.N.); fakher.chabchoub@yahoo.fr (F.C.); zfnfethi@yahoo.fr (F.Z.); 2 Laboratory of Medicinal Chemistry of Natural Substances and Redox Pharmacophores, University of Toulouse, UPS; UMR 152, F-31062 Toulouse cedex 9, France; E-Mails: sudor@cict.fr (J.S.); romain@cict.fr (R.D.); 3 IRD, UMR 152, F-31062 Toulouse cedex 9, France

**Keywords:** 2(1*H*)-pyrimidinones, 1,2,3-triazoles, (3 + 2) cycloaddition, alkynes, azides

## Abstract

In this paper we present the room temperature synthesis of a novel serie of 1,4-disubstituted-1,2,3-triazoles **4a-l** by employing the (3 + 2) cycloaddition reaction of pyrimidinones containing alkyne functions with different model azides in the presence of copper sulphate and sodium ascorbate. To obtain the final triazoles, we also synthesized the major precursors 6-amino-5-cyano-1,4-disubstituted-2(1*H*)-pyrimidinones **3a-r** from ethyl 2,2-dicyanovinylcarbamate derivatives **2a-c** and various primary aromatic amines containing an alkyne group. The triazoles were prepared in good to very good yields.

## Introduction

The copper-(I)-catalyzed Huisgen–Sharpless–Meldal 1,3-dipolar cycloaddition between alkynes and azides (‘click’ chemistry) resulting in the formation of 1,4-disubstituted-1,2,3-triazoles has gained significant importance because of its wide range of applications in various fields of drug discovery [1], bioconjugation [2] and material or surface science [3,4]. Amongst the various classes of nitrogen heterocycles, 1,2,3-triazoles and their derivatives deserve special recognition due to their wide usage in industrial applications as dyes, photographic materials, corrosion inhibitors and as herbicidal, fungicidal and antibacterial agrochemicals [5,6]. Several members of the 1,2,3-triazole family exhibit a broad spectrum of antiinfectious properties such as antimicrobial [7], anti-HIV [8], anti-allergic [9] and antimalarial activities [10]. On the other hand, 2(1*H*)-pyrimidinones also show significant biological activities [11]. For instance, 2(1*H*)-pyrimidinones derivatives have been screened for antihypertension [12], insulin-mimetic [13], anti-inflammatory [14] and anti-proliferative [15] activities or as selective α_1a_-andrenergic receptor antagonists [16]. Interested by the wide variety of pharmacological properties and potential applications of both 2(1*H*)-pyrimidinones and 1,2,3-triazoles we have designed the synthesis of hybrid molecules consisting of both moieties. Our method is based on the (3 + 2) cycloaddition reaction of 6-amino-5-cyano-1-(*meta*- or *para*-ethynylphenyl)-4-substituted-2(1*H*)-pyrimidinones with different azides in the presence of copper sulphate and sodium ascorbate at room temperatures that affords 1,4-disubstituted-1,2,3-triazoles.

## Results and Discussion

**Scheme 1:**
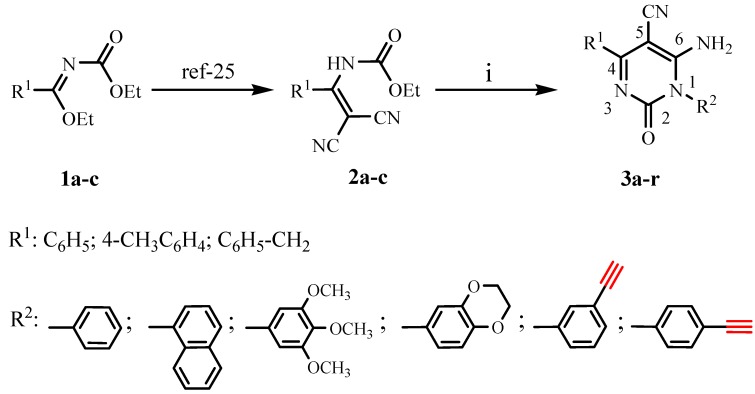
Synthesis of 6-amino-5-cyano-1,4-disubstituted-2(1*H*)-pyrimidinones (**3a-r**). Reagents and conditions: (i) primary aromatic amines, chlorobenzene, 110 °C, (2~4) h.

Ethyl 2,2-dicyanovinylcarbamate derivatives **2a-c** were prepared in good yields by action of malononitrile with ethyl *N-*(ethoxycarbonyl)imidates **1a-c** following a previously reported method [17] (Scheme 1). The reaction of these compounds **2a-c** with primary aromatic amines in chlorobenzene under reflux yielded the 6-amino-5-cyano-1,4-disubstituted-2(1*H*)-pyrimidinones **3a-r** in yields ranging from 55 to 76% (Table 1). This synthetic method is more general and easier to implement than the methods already described in the literature [18,19].

**Table 1:** Synthesis of 6-amino-5-cyano-1,4-disubstituted-2(1*H*)-pyrimidinones **3a-r**.

Entry	**Compound**	R^1^	R^2^	Yields^a^
1	**3a^26^**	Ph	Phenyl	75%
2	**3b**	Ph	Naphthalen-1-yl	68%
3	**3c**	Ph	3,4,5-Trimethoxyphenyl	71%
4	**3d**	Ph	2,3-Dihydrobenzo[*b*][1,4]dioxin-6-yl	74%
5	**3e**	Ph	3-Ethynylphenyl	62%
6	**3f**	Ph	4-Ethynylphenyl	60%
7	**3g^26^**	4-CH_3_Ph	Phenyl	73%
8	**3h**	4-CH_3_Ph	Naphthalen-1-yl	67%
9	**3i**	4-CH_3_Ph	3,4,5-Trimethoxyphenyl	75%
10	**3j**	4-CH_3_Ph	2,3-Dihydrobenzo[*b*][1,4]dioxin-6-yl	72%
11	**3k**	4-CH_3_Ph	3-Ethynylphenyl	70%
12	**3l**	4-CH_3_Ph	4-Ethynylphenyl	61%
13	**3m**	Ph-CH_2_	Phenyl	76%
14	**3n**	Ph-CH_2_	Naphthalen-1-yl	59%
15	**3o**	Ph-CH_2_	3,4,5-Trimethoxyphenyl	65%
16	**3p**	Ph-CH_2_	2,3-Dihydrobenzo[*b*][1,4]dioxin-6-yl	55%
17	**3q**	Ph-CH_2_	3-Ethynylphenyl	62%
18	**3r**	Ph-CH_2_	4-Ethynylphenyl	58%

^a^ Isolated yield.

**Scheme 2:**
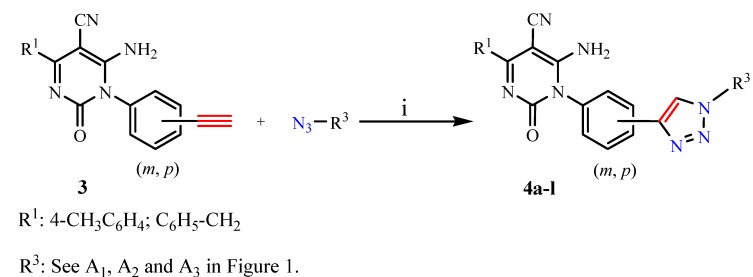
Synthesis of 1,4-disubstituted-1,2,3-triazoles **4a-l**. Reagents and conditions: (a) Na-ascorbate (0.45 equiv), CuSO4·5H_2_O (0.1 equiv), THF/H_2_O/*t*-BuOH (3:1:1, v/v/v), rt, 2d.

**Figure 1:**
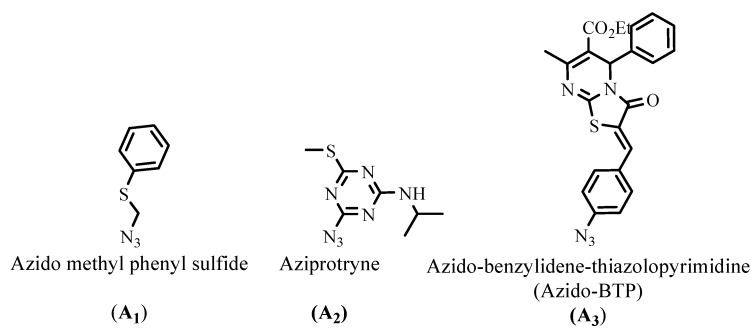
Structures of the three different azides used in this work.

The (3 + 2) cycloaddition of 6-amino-5-cyano-1-(*meta*- or *para*-ethynylphenyl)-4-substituted-2(1*H*)-pyrimidinones **3k**, **3l**, **3q** and **3r** with different azides **A_1_**, **A_2_** and **A_3_** (Figure 1) in the presence of Na-ascorbate, THF/*t*-BuOH/H_2_O and CuSO_4_**.**5H_2_O, at room temperature resulted in the corresponding 1,4-disubstituted-1,2,3-triazole compounds **4a-l** (Scheme 2) in good yields (Table 2). The structures of compounds **3a-r** were in accordance with their spectroscopic data. The IR spectra of the compounds in general exhibited an absorption band at 2,210 cm^−1^ indicating the presence of one cyano group. The absorption band at around 3,265–3,275 cm^−1^ for the compounds **3e**, **3f**, **3k**, **3l**, **3q** and **3r** indicated that the terminal alkyne C≡C-H was present in these compounds.

**Table 2:** Synthesis of 1,4-disubstituted-1,2,3-triazoles **4a-l**.

Entry	**Compound**	Alkynes	Azides	Yields^a^
1	**4a**	3k	A_1_	82%
2	**4b**	3l	A_1_	72%
3	**4c**	3q	A_1_	80%
4	**4d**	3r	A_1_	75%
5	**4e**	3k	A_2_	73%
6	**4f**	3l	A_2_	94%
7	**4g**	3q	A_2_	76%
8	**4h**	3r	A_2_	71%
9	**4i**	3k	A_3_	84%
10	**4j**	3l	A_3_	72%
11	**4k**	3q	A_3_	81%
12	**4l**	3r	A_3_	88%

^a^ Isolated yield.

The mass spectra showed the respective [M + H]^+^ peaks. In the ^1^H-NMR spectra the most significant information was the disappearance of triplet and quadruplet of ethoxy groups present in the starting reagent **2a-c** and the appearance of signals for the protons of the group **R**^2^ introduced by the primary aromatic amines. 

Structures of compounds **4a-l** were established on the basis of their spectroscopic data. The IR absorption band corresponding to a terminal C≡C-H group was not observed around 3,271 cm^−1^. The mass spectra showed the respective [M + H]^+^ peaks. According to ^1^H-NMR spectra of the ‘click’ products the terminal triple bonded proton signal (δH = 4.3 ppm) of the alkynes **3** disappeared and the newly formed triazole signal was observed at 8.5–9.5 ppm. The triazole ring formation was also identified from the ^13^C-NMR spectra with the new signals of the ethylenic C atoms of the 1,2,3-triazole moiety at δ = 120–122 ppm (CH_ar-triazole_) and δ = 146–148 ppm (C_q-triazole_).

### X-ray crystal analysis of compounds 3b and ***3g***

**Figure 2:**
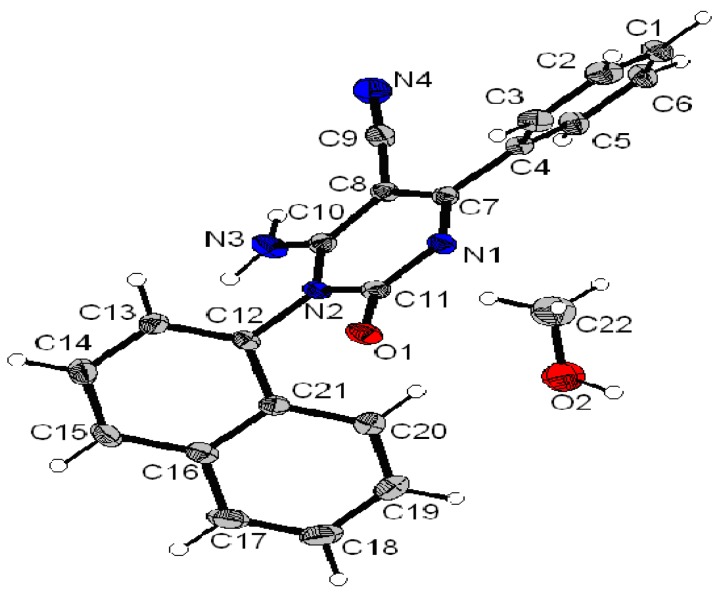
X-ray crystal analysis of compound **3b**.

**Figure 3:**
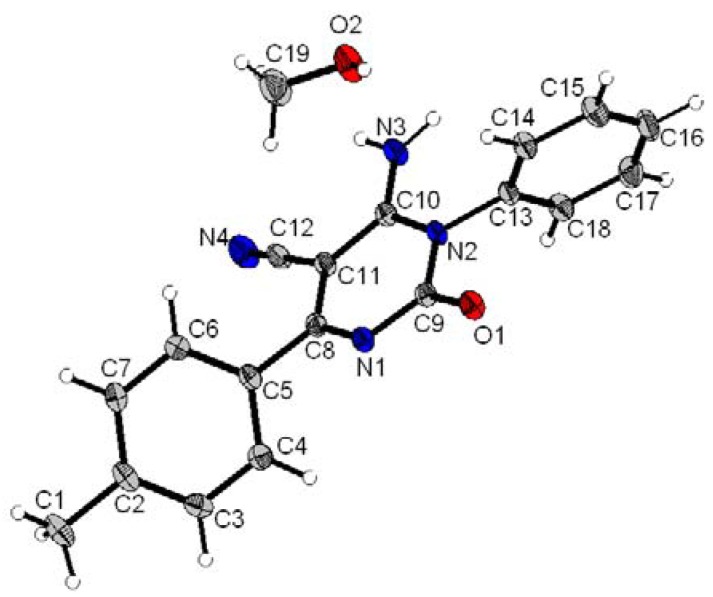
X-ray crystal analysis of compound **3g**.

To further confirm the structure of compounds **3**, an X-ray crystallographic study of compounds **3b** and **3g** was carried out (Figure 2 and Figure 3). Crystals were obtained by slow evaporation from methanol solution. Crystallographic data were collected at 180K with an Oxford-Diffraction XCALIBUR CCD Diffractometer equipped with a Cryojet cooler device from Oxford Instruments. Structures were solved by direct methods using SIR92 [20] and refined by full-matrix least-squares procedures on F using the programs of the PC version of CRYSTALS [21]. Atomic scattering factors were taken from the International Tables for X-ray Crystallography [22]. 

*Data for* **3b**: C_21_H_14_N_4_O, CH_4_O, M = 370.41, colorless block crystal, 0.15 × 0.20 × 0.25 mm^3^, monoclinic, space group P 2_1_/c, a = 11.1402(4), b = 19.9585(6), c = 8.4770(3) Å, β = 105.839(4)°, V = 1813.23(11) Å^3^, Z = 4, d = 1.36, µ(MoKα) = 0.090, 253 parameters, 17,459 reflexions measured, 4,850 unique (R int = 0.030), 3,133 reflections used in the calculations (I > 3σ[I]), R = 0.0345, wR = 0.0403, residual electronic density = - 0.17/0.32 (e.Å^-^^3^).

*Data for* **3g**: C_18_H_14_N_4_O, CH_4_O, M = 334.38, colorless block crystal, 0.20 x 0.20 x 0.20 mm^3^, monoclinic, space group P 2_1_/c, a = 10.5511(3), b = 14.2950(5), c = 10.9120(4) Å, β = 93.184(3)°, V = 1643.29(10) Å^3^, Z = 4, d = 1.35, µ(MoKα) = 0.091, 226 parameters, 14,902 reflexions measured, 4,408 unique (R int = 0.030), 2,930 reflections used in the calculations (I > 3σ[I]), R = 0.0428, wR = 0.0462, residual electronic density = - 0.22/0.39 (e.Å^-^^3^).

CCDC contain the supplementary crystallographic data for this paper. These data can be obtained free of charge from The Cambridge Crystallographic Data Centre via www.ccdc.cam.ac.uk/data_request/cif.

## Conclusions

In summary, various 6-amino-5-cyano-1-(*meta*- or *para*-ethynylphenyl)-4-substituted-2(1*H*)-pyrimidinones were synthesized and utilized as starting materials in the ‘click’ reaction to attach azido residues. Consequently, we have employed these, in house synthesized precursors, to prepare a new class of hybrid molecules 1,4-disubstituted-1,2,3-triazoles employing already known chemistry of (3 + 2) cycloaddition of azides and acetylenes in good to very good yields. All products that we have obtained were hitherto unknown. A number of them are presently under pharmacological screening.

## Experimental

Commercially available reagent grade chemicals were used as received without additional purification. All reactions were followed by TLC (E. Merck Kieselgel 60 F-254), with detection by UV light at 254 nm. Column chromatography was performed on silica gel (60–200 mesh E. Merck). IR spectra were recorded on a Perkin-Elmer PARAGON 1000 FT-IR spectrometer. ^1^H- and ^13^C-NMR spectra were recorded on an AC Bruker spectrometer at 300 MHz (^1^H) and 75 MHz (^13^C) using (CD_3_)_2_SO as solvent with (CD_3_)_2_SO (δ_H_ 2.5) or (CD_3_)_2_SO (δ_C_ 39.5). Chemical shifts (δ) are reported in parts per million (ppm) relative to tetramethylsilane (0 ppm) as internal reference and the following multiplicity abbreviations were used: s, singlet; d, doublet; t, triplet; q, quadruplet; m, multiplet; *J* in hertz. The mass spectra were recorded on an ion trap mass spectrometer (Finnigan LCQ Deca XP Max) using electrospray as an ionization source. Melting points were determined on an Electrothermal 9300 capillary melting point apparatus and are uncorrected. UV-visible spectra were recorded on a Specord 205 Analytikjena spectrophotometer. The purity of all compounds was determined by LC-PDA-MS methods and was found to be in the range between 96–99%. 

### General experimental procedure for preparation of 6-amino-5-cyano-1,4-disubstituted-2(1H)-pyrimid-inones ***3a-r***

To a magnetically stirred solution of the ethyl 2,2-dicyanovinylcarbamate derivatives **2a-c** (1 equiv) in chlorobenzene (25 mL), a primary aromatic amine (1.2 equiv) added and reaction mixture was stirred for 2~4 h at 110 °C. Reaction progress was monitored by TLC using the indicated eluents. The resulting mixture was allowed to cool at room temperature. The formed precipitate was isolated by filtration and washed with ethanol or with diethyl ether for **3e**, **3f**, **3k**, **3l**, **3q** and **3r** to give pure products.

*6-Amino-5-cyano-1,4-diphenyl-2(1H)-pyrimidinone* (**3a**). White cristals, yield (75%), C_17_H_12_N_4_O, M = 288 gmol^−1^, mp 252–254 °C, R*_f_* = 0.21 (ethyl acetate/dichloromethane, 70:30, v/v); UV (MeOH) λ_max_ nm (ε Lmol^−1^cm^−1^): 248 (32,400), 318 (11,232); IR (KBr) cm^−1^: 3,450–3,310 (NH_2_), 2,212 (CN), 1,665 (C=O), 1,616 (C=N); ^1^H-NMR: (DMSO-d_6_): δ = 7.34–7.82 (m, 12H, Ar-H + NH_2_); ^13^C-NMR (DMSO-d_6_): δ = 72.9 (C-5), 117.1 (CN), 128.8, 128.9, 130, 130.7, 131.5, 135.1, 137.5, 154.1 (C-2), 160.5 (C-4), 172 (C-6); MS-(+)ESI: *m/z* (%): 599 ([2M+Na]^+^, 35), 311 ([M+Na]^+^, 4), 289 ([M+H]^+^, 100). 

*6-Amino-5-cyano-1-(naphthalen-1-yl)-4-phenyl-2(1H)-pyrimidinone* (**3b**). Greyish white solid, yield (68%), C_21_H_14_N_4_O, M = 338 gmol^−1^, mp 198–200 °C, R*_f_* = 0.54 (ethyl acetate/dichloromethane, 70:30, v/v); UV (MeOH) λ_max_ nm (ε Lmol^−1^cm^−1^): 248 (37,518), 318 (14,196); IR (KBr) cm^−1^: 3,450–3,310 (NH_2_), 2,211 (CN), 1,672 (C=O), 1,620 (C=N); ^1^H-NMR: (DMSO-d_6_): δ = 7.42–8.15 (m, 14H, Ar-H + NH_2_); ^13^C-NMR (DMSO-d_6_): δ = 73.1 (C-5), 117.2 (CN), 122, 127.1, 127.2, 127.8, 128, 128.8, 129, 129.2, 129.7, 130.7, 131.5, 131.6, 135.1, 137.6, 154.2 (C-2), 160.9 (C-4), 172.6 (C-6); MS-(+)ESI: *m/z* (%): 699 ([2M+Na]^+^, 16), 339 ([M+H]^+^, 100).

*6-Amino-5-cyano-1-(3,4,5-trimethoxyphenyl)-4-phenyl-2(1H)-pyrimidinone* (**3c**). Yellowish white solid, yield (71%), C_20_H_18_N_4_O_4_, M = 378 gmol^−1^, mp 255–257 °C, R*_f_* = 0.21 (ethyl acetate/dichloromethane, 70:30, v/v); UV (MeOH) λ_max_ nm (ε L.mol^−1^cm^−1^): 249 (51,030), 320 (14,742); IR (KBr) cm^−1^: 3,450-3,310 (NH_2_), 2,209 (CN), 1,687 (C=O), 1,620 (C=N); ^1^H-NMR: (DMSO-d_6_): δ = 3.74 (s, 6H, 2OCH_3_), 3.76 (s, 3H, OCH_3_), 7.39–7.60 (m, 9H, Ar-H + NH_2_); ^13^C-NMR (DMSO-d_6_): δ = 56.5 (2C, 2OCH_3_), 60.4 (OCH_3_), 72.6 (C-5), 106.4, 117.1 (CN), 125.9, 128.6, 130.5, 136.9, 138, 138.4, 153.9, 154 (C-2), 160.7 (C-4), 172 (C-6); MS-(+)ESI: *m/z* (%): 779 ([2M+Na]^+^, 3), 379 ([M+H]^+^, 100).

*6-Amino-5-cyano-1-(2,3-dihydrobenzo[b]*[1,4]*dioxin-6-yl)-4-phenyl-2(1H)-pyrimidinone* (**3d**). Pale brown solid, yield (74%), C_19_H_14_N_4_O_3_, M = 346 gmol^−1^, mp 283–285 °C, R*_f_* = 0.49 (ethyl acetate/dichloromethane, 70:30, v/v); UV (MeOH) λ_max_ nm (ε Lmol^−1^cm^−1^): 248 (37887), 318 (12456); IR (KBr) cm^−1^: 3,450-3,310 (NH_2_), 2,210 (CN), 1,696 (C=O), 1,662 (C=N); ^1^H-NMR: (DMSO-d_6_): δ = 4.30 (s, 4H, 2CH_2_), 6.80-7.59 (m, 10H, Ar-H + NH_2_); ^13^C-NMR (DMSO-d_6_): δ = 64.5 (CH_2_), 64.6 (CH_2_), 72.6 (C-5), 117.2 (CN), 118.5, 121.7, 125.7, 127.6, 129.2, 131.9, 137.7, 138.1, 144.5, 144.6, 154.1 (C-2), 160.7 (C-4), 171.8 (C-6); MS-(+)ESI: *m/z* (%): 715 ([2M+Na]^+^, 8), 347 ([M+H]^+^, 100).

*6-Amino-5-cyano-1-(3-ethynylphenyl)-4-phenyl-2(1H)-pyrimidinone* (**3e**). Pale yellow solid, yield (62%), C_19_H_12_N_4_O, M = 312 gmol^−1^, mp 237–239 °C, R*_f_* = 0.52 (ethyl acetate/dichloromethane, 50:50, v/v); UV (MeOH) λ_max_ nm (ε Lmol^−1^cm^−1^): 254 (36,972), 307 (14,508); IR (KBr) cm^−1^: 3,450–3,310 (NH_2_), 3,270 (≡C-H), 2,209 (CN), 1,665 (C=O), 1,636 (C=N); ^1^H-NMR: (DMSO-d_6_): δ = 4.32 (s, 1H, C≡CH), 7.27–7.86 (m, 11H, Ar-H + NH_2_); ^13^C-NMR (DMSO-d_6_): δ = 73.7 (C-5), 82.4 (C≡CH), 83.2 (C≡CH), 117.2 (CN), 124.1, 128.6, 129.2, 129.9, 131.1, 132.4, 133.4, 134.5, 135.4, 141.5, 154 (C-2), 160.5 (C-4), 171.7 (C-6); MS-(+)ESI: *m/z* (%): 647 ([2M+Na]^+^, 21), 313 ([M+H]^+^, 100).

*6-Amino-5-cyano-1-(4-ethynylphenyl)-4-phenyl-2(1H)-pyrimidinone* (**3f**). White solid, yield (60%), C_19_H_12_N_4_O, M = 312 gmol^−1^, mp 206–208 °C, R*_f_* = 0.57 (ethyl acetate/dichloromethane, 50:50, v/v); UV (MeOH) λ_max_ nm (ε Lmol^−1^cm^−1^): 247 (38,844), 309 (13,572); IR (KBr) cm^−1^: 3,450-3,310 (NH_2_), 3,268 (≡C-H), 2,210 (CN), 1,676 (C=O), 1,635 (C=N); ^1^H-NMR: (DMSO-d_6_): δ = 4.31 (s, 1H, C≡CH), 7.34–7.91 (m, 11H, Ar-H + NH_2_); ^13^C-NMR (DMSO-d_6_): δ = 73.7 (C-5), 82.3 (C≡CH), 83.2 (C≡CH), 117.2 (CN), 125, 128.5, 130.1, 131.2, 132.4, 133.4, 135.4, 141.3, 154 (C-2), 160 (C-4), 172.1 (C-6); MS-(+)ESI: *m/z* (%): 647 ([2M+Na]^+^, 10), 313 ([M+H]^+^, 100).

*6-Amino-5-cyano-4-(4-methylphenyl)-1-phenyl-2(1H)-pyrimidinone* (**3g**). White solid, yield (73%), C_18_H_14_N_4_O, M = 302 gmol^−1^, mp 257–259 °C, R*_f_* = 0.47 (ethyl acetate/dichloromethane, 70:30, v/v);UV (MeOH) λ_max_ nm (ε Lmol^−1^cm^−1^): 250 (26,727), 319 (11,325); IR (KBr) cm^−1^: 3,450–3,310 (NH_2_), 2,211 (CN), 1,674 (C=O), 1,619 (C=N); ^1^H-NMR: (DMSO-d_6_): δ = 2.52 (s, 3H, CH_3_), 7,45–7,9 (m, 11H, Ar-H + NH_2_); ^13^C-NMR (DMSO-d_6_): δ = 21.5 (CH_3_), 72.6 (C-5), 118.3 (CN), 128.8, 129, 130.1, 130.8, 131.5, 132.4, 135.1, 137.5, 154.3 (C-2), 160.8 (C-4), 172.5 (C-6); MS-(+)ESI: *m/z* (%): 627 ([2M+Na]^+^, 31), 325 ([M+Na]^+^, 3), 303 ([M+H]^+^, 100). 

*6-Amino-5-cyano-4-(4-methylphenyl)-1-(naphthalen-1-yl)-2(1H)-pyrimidinone* (**3h**). Greyish green solid, yield (67%), C_22_H_16_N_4_O, M = 352 gmol^−1^, mp 261–263 °C, R*_f_* = 0.57 (ethyl acetate/ dichloromethane, 50:50, v/v); UV (MeOH) λ_max_ nm (ε Lmol^−1^cm^−1^): 251 (26,928), 318 (12,672); IR (KBr) cm^−1^: 3,450-3,310 (NH_2_), 2,209 (CN), 1,686 (C=O), 1,617 (C=N); ^1^H-NMR: (DMSO-d_6_): δ = 2.43 (s, 3H, CH_3_), 7.38-8.15 (m, 13H, Ar-H + NH_2_); ^13^C-NMR (DMSO-d_6_): δ = 21.5 (CH_3_), 72.4 (C-5), 118.3 (CN), 122.5, 127, 127.2, 127.9, 128.1, 128.9, 129.1, 129.3, 129.8, 130.7, 131.4, 132.1, 135.6, 137.7, 154.6 (C-2), 161 (C-4), 172.8 (C-6); MS-(+)ESI: *m/z* (%): 727 ([2M+Na]^+^, 15), 353 ([M+H]^+^, 100).

*6-Amino-5-cyano-4-(4-methylphenyl)-1-(3,4,5-trimethoxyphenyl)-2(1H)-pyrimidinone* (**3i**). Pale yellow solid, yield (75%), C_21_H_20_N_4_O_4_, M = 392 gmol^−^^1^, mp 292–294 °C, R*_f_* = 0.53 (ethyl acetate/ dichloromethane, 70:30, v/v); UV (MeOH) λ_max_ nm (ε Lmol^−^^1^cm^−^^1^): 250 (52,920); IR (KBr) cm^−^^1^: 3,450-3,310 (NH_2_), 2,212 (CN), 1,671 (C=O), 1,625 (C=N); ^1^H-NMR: (DMSO-d_6_): δ = 2.41 (s, 3H, CH_3_), 3.74 (s, 6H, 2OCH_3_), 3.77 (s, 3H, OCH_3_), 7.4–7.59 (m, 8H, Ar-H + NH_2_); ^13^C-NMR (DMSO-d_6_): δ = 21.4 (CH_3_), 56.6 (2C, 2OCH_3_), 60.4 (OCH_3_), 72.6 (C-5), 106.4, 117.1 (CN), 125.8, 128.6, 130.5, 137.5, 138.1, 138.5, 154, 154.5 (C-2), 160.6 (C-4), 171.9 (C-6); MS-(+)ESI: *m/z* (%): 393 ([M+H]^+^, 100).

*6-Amino-5-cyano-1-(2,3-dihydrobenzo[b]*[1,4]*dioxin-6-yl)-4-(4-methylphenyl)-2(1H)-pyrimidinone* (**3j**). Yellow solid, yield (72%), C_20_H_16_N_4_O_3_, M = 360 gmol^−^^1^, mp 284–286 °C, R*_f_* = 0.54 (ethyl acetate/dichloromethane, 70:30, v/v); UV (MeOH) λ_max_ nm (ε Lmol^−^^1^cm^−^^1^): 251 (32,940), 280 (19,980), 318 (13,500); IR (KBr) cm^−^^1^: 3,450–3,310 (NH_2_), 2,211 (CN), 1,658 (C=O), 1,634 (C=N); ^1^H-NMR: (DMSO-d_6_): δ = 2.40 (s, 3H, CH_3_), 4.30 (s, 4H, 2CH_2_), 6.79-7.60 (m, 9H, Ar-H + NH_2_); ^13^C- NMR (DMSO-d_6_): δ = 21.4 (CH_3_), 64.5 (CH_2_), 64.6 (CH_2_), 72.6 (C-5), 117.1 (CN), 117.9, 121.5, 125.8, 127.7, 128.5, 132, 137.4, 138, 144.8, 144.8, 154.1 (C-2), 160.7 (C-4), 171.8 (C-6); MS-(+)ESI: *m/z* (%): 743 ([2M+Na]^+^, 9), 361 ([M+H]^+^, 100).

*6-Amino-5-cyano-1-(3-ethynylphenyl)-4-(4-methylphenyl)-2(1H)-pyrimidinone* (**3k**). Pale yellow solid, yield (70%), C_20_H_14_N_4_O, M = 326 gmol^−^^1^, mp 247–249 °C, R*_f_* = 0.61 (ethyl acetate/dichloromethane, 50:50, v/v); UV (MeOH) λ_max_ nm (ε Lmol^−^^1^cm^−^^1^): 252 (24,339), 307 (16,137); IR (KBr) cm^−^^1^: 3,450–3,310 (NH_2_), 3,271 (≡C-H), 2,211 (CN), 1,671 (C=O), 1,640 (C=N); ^1^H-NMR: (DMSO-d_6_): δ = 2.41 (s, 3H, CH_3_), 4.32 (s, 1H, C≡CH), 7.25–7.75 (m, 10H, Ar-H + NH_2_); ^13^C-NMR (DMSO-d_6_): δ = 21.5 (CH_3_), 73.81 (C-5), 82.4 (C≡CH), 83.2 (C≡CH), 117.2 (CN), 124, 128.8, 129.3, 129.8, 131, 132.5, 133.3, 134.6, 135.6, 141.5, 153.9 (C-2), 160.5 (C-4), 171.8 (C-6); MS-(+)ESI: *m/z* (%): 675 ([2M+Na]^+^, 19), 327 ([M+H]^+^, 100).

*6-Amino-5-cyano-1-(4-ethynylphenyl)-4-(4-methylphenyl)-2(1H)-pyrimidinone* (**3l**). Brownish yellow solid, yield (61%), C_20_H_14_N_4_O, M = 326 gmol^−^^1^, mp 192–194 °C, R*_f_* = 0.49 (ethyl acetate/ dichloromethane, 50:50, v/v); UV (MeOH) λ_max_ nm (ε Lmol^−^^1^cm^−^^1^): 250 (28,851), 308 (15,159); IR (KBr) cm^−^^1^: 3,450–3,310 (NH_2_), 3,270 (≡C-H), 2,209 (CN), 1,662 (C=O), 1,638 (C=N); ^1^H-NMR: (DMSO-d_6_): δ = 2.41 (s, 3H, CH_3_), 4.30 (s, 1H, C≡CH), 7.27–7.94 (m, 10H, Ar-H + NH_2_); ^13^C-NMR (DMSO-d_6_): δ = 21.5 (CH_3_), 72.9 (C-5), 82.3 (C≡CH), 83.3 (C≡CH), 117.2 (CN), 124.1, 128.9, 129.3, 131.1, 132.5, 134.4, 135.8, 141.3, 153.9 (C-2), 160.1 (C-4), 171.6 (C-6); MS-(+)ESI: *m/z* (%): 675 ([2M+Na]^+^, 9), 327 ([M+H]^+^, 100).

*6-Amino-4-benzyl-5-cyano-1-phenyl-2(1H)-pyrimidinone* (**3m**). White solid, yield (76%), C_18_H_14_N_4_O, M = 302 gmol^−1^, mp 276–278 °C, R*_f_* = 0.31 (ethyl acetate/dichloromethane, 70:30, v/v); UV (MeOH) λ_max_ nm (ε Lmol^−1^cm^−1^): 248 (22,197), 308 (14,043); IR (KBr) cm^−1^: 3,450-3,310 (NH_2_), 2,211 (CN), 1,680 (C=O), 1,614 (C=N); ^1^H-NMR: (DMSO-d_6_): δ = 3.9 (s, 2H, CH_2_), 7.3–7.53 (m, 12H, Ar-H + NH_2_); ^13^C-NMR (DMSO-d_6_): δ = 43.4 (CH_2_), 73.7 (C-5), 116.7 (CN), 127.3, 128.9, 129, 129.4, 129.7, 130.7, 135, 137.1, 154.1 (C-2), 159.7 (C-4), 175.4 (C-6); MS-(+)ESI: *m/z* (%): 627 ([2M+Na]^+^, 20), 325 ([M+Na]^+^, 3), 303 ([M+H]^+^, 100).

*6-Amino-4-benzyl-5-cyano-1-(naphthalen-1-yl)-2(1H)-pyrimidinone* (**3n**). Pale violet solid, yield (59%), C_22_H_16_N_4_O, M = 352 gmol^−1^, mp 252–254 °C, R*_f_* = 0.47 (ethyl acetate/dichloromethane, 50:50, v/v); UV (MeOH) λ_max_ nm (ε Lmol^−1^cm^−1^): 248 (24,288), 296 (17,952); IR (KBr) cm^−1^: 3,450–3,310 (NH_2_), 2,212 (CN), 1,678 (C=O), 1,618 (C=N); ^1^H-NMR: (DMSO-d_6_): δ = 3.97 (s, 2H, CH_2_), 7.27–8.27 (m, 14H, Ar-H + NH_2_); ^13^C-NMR (DMSO-d_6_): δ = 43.6 (CH_2_), 73.7 (C-5), 116.6 (CN), 117.9, 121.7, 126.5, 126.9, 127.4, 127.8, 127.9, 129.1, 129.6, 129.7, 131.3, 134.3, 135, 137.1, 154.1 (C-2), 160 (C-4), 175.9 (C-6); MS-(+)ESI: *m/z* (%): 727 ([2M+Na]^+^, 10), 353 ([M+H]^+^, 100).

*6-Amino-4-benzyl-5-cyano-1-(3,4,5-trimethoxyphenyl)-2(1H)-pyrimidinone* (**3o**). Yellow solid, yield (65%), C_21_H_20_N_4_O_4_, M = 392 gmol^−1^, mp 224–226 °C, R*_f_* = 0.26 (ethyl acetate/dichloromethane, 70:30, v/v); UV (MeOH) λ_max_ nm (ε L.mol^−1^cm^−1^): 247 (39,396), 307 (19,404); IR (KBr) cm^−1^: 3,450–3,310 (NH_2_), 2,208 (CN), 1,687 (C=O), 1,627 (C=N); ^1^H-NMR: (DMSO-d_6_): δ = 3.91 (s, 2H, CH_2_), 3.75 (s, 6H, 2OCH_3_), 3.77 (s, 3H, OCH_3_), 7.47–8.62 (m, 9H, Ar-H + NH_2_); ^13^C-NMR (DMSO-d_6_): δ = 43.4 (CH_2_), 56.6 (2C, 2OCH_3_), 60.4 (OCH_3_), 72.7 (C-5), 107.1, 116.9 (CN), 126, 128.6, 129.9, 137.1, 138.3, 138.5, 153.6, 154.4 (C-2), 160.7 (C-4), 175 (C-6); MS-(+)ESI: *m/z* (%): 393 ([M+H]^+^, 100).

*6-Amino-4-benzyl-5-cyano-1-(2,3-dihydrobenzo[b]*[1,4]*dioxin-6-yl)-2(1H)-pyrimidinone* (**3p**). Dark brown solid, yield (55%), C_20_H_16_N_4_O_3_, M = 360 gmol^−1^, mp 287–289 °C, R*_f_* = 0.41 (ethyl acetate/ dichloromethane, 70:30, v/v); UV (MeOH) λ_max_ nm (ε Lmol^−1^cm^−1^): 251 (31,320), 318 (15,120); IR (KBr) cm^−1^: 3,450-3,310 (NH_2_), 2,210 (CN), 1,661 (C=O), 1,609 (C=N); ^1^H-NMR: (DMSO-d_6_): δ = 3.9 (s, 2H, CH_2_), 4.32 (s, 4H, 2CH_2_), 6.79–8.05 (m, 10H, Ar-H + NH_2_); ^13^C-NMR (DMSO-d_6_): δ = 43.4 (CH_2_), 64.5 (CH_2_), 64.8 (CH_2_), 72.6 (C-5), 116.9 (CN), 117.4, 121.7, 126, 127.5, 128.1, 131.5, 136.9, 138.1, 143.9, 144.2, 153.9 (C-2), 160.5 (C-4), 175.4 (C-6); MS-(+)ESI: *m/z* (%): 743 ([2M+Na]^+^, 11), 361 ([M+H]^+^, 100).

*6-Amino-4-benzyl-5-cyano-1-(3-ethynylphenyl)-2(1H)-pyrimidinone* (**3q**). White solid, yield (62%), C_20_H_14_N_4_O, M = 326 gmol^−1^, mp 224–226 °C, R*_f_* = 0.47 (ethyl acetate/dichloromethane, 50:50, v/v); UV (MeOH) λ_max_ nm (ε Lmol^−1^cm^−1^): 248 (40,587), 308 (15,160); IR (KBr) cm^−1^: 3,450–3,310 (NH_2_), 3,271 (≡C-H), 2,209 (CN), 1,662 (C=O), 1,625 (C=N); ^1^H-NMR: (DMSO-d_6_): δ = 3.9 (s, 2H, CH_2_), 4.3 (s, 1H, C≡CH), 7.32–7.83 (m, 11H, Ar-H + NH_2_); ^13^C-NMR (DMSO-d_6_): δ = 43.4 (CH_2_), 73.8 (C-5), 82.3 (C≡CH), 83.2 (C≡CH), 116.5 (CN), 123.9, 128.5, 129.2, 130.2, 131.4, 132.7, 132.9, 134.3, 136.4, 142.1, 154.1 (C-2), 160.8 (C-4), 174.9 (C-6); MS-(+)ESI: *m/z* (%): 675 ([2M+Na]^+^, 17), 327 ([M+H]^+^, 100).

*6-Amino-4-benzyl-5-cyano-1-(4-ethynylphenyl)-2(1H)-pyrimidinone* (**3r**). White solid, yield (58%), C_20_H_14_N_4_O, M = 326 gmol^−1^, mp 252–254 °C, R*_f_* = 0.56 (ethyl acetate/dichloromethane, 50:50, v/v); UV (MeOH) λ_max_ nm (ε Lmol^−1^cm^−1^): 250 (44,010), 318 (12,714); IR (KBr) cm^−1^: 3,450–3,310 (NH_2_), 3,269 (≡C-H), 2,211 (CN), 1,668 (C=O), 1,639 (C=N); ^1^H-NMR: (DMSO-d_6_): δ = 3.89 (s, 2H, CH_2_), 4.31 (s, 1H, C≡CH), 7.27–7.63 (m, 11H, Ar-H + NH_2_); ^13^C-NMR (DMSO-d_6_): δ = 43.4 (CH_2_), 73.8 (C-5), 82.3 (C≡CH), 83.4 (C≡CH), 116.5 (CN), 123.4, 127.2, 128.9, 129.5, 129.6, 133.9, 135.5, 137, 153.9 (C-2), 159.6 (C-4), 175.5 (C-6); MS-(+)ESI: *m/z* (%): 675 ([2M+Na]^+^, 8), 327 ([M+H]^+^, 100).

### General experimental procedure for preparation of 1,4-disubstituted-1,2,3-triazoles ***4a-l***

The mixture of alkyne **3** (1 mmol) and azides (1 mmol) was suspended in a mixture of THF/*t*-BuOH/H_2_O (3:1:1, v/v/v, 6/2/2 mL). Sodium ascorbate (89 mg, 0.45 equiv) was added followed by addition of CuSO_4_5H_2_O (16 mg, 0.1 equiv). The heterogeneous mixture was stirred vigorously for 2 days, at which time TLC showed complete conversion. The reaction mixture was concentrated under vacuum and the residue was treated with H_2_O (50 mL) and extracted with dichloromethane (3 × 15 mL). The combined organic extracts were dried over anhydrous Na_2_SO_4_, filtered and evaporated under reduced pressure to give a crude mass. Column chromatography purification using ethyl acetate/dichloromethane as eluent gave the clicked product **4**.

*6-Amino-5-cyano-4-(4-methylphenyl)-1-(3-(1-(phenylthiomethyl)-1H-1,2,3-triazol-4-yl)phenyl)-2(1H)-pyrimidinone* (**4a**). White solid, yield (82%), C_27_H_21_N_7_OS, M = 491 gmol^−^^1^, mp 220–222 °C, R*_f_* = 0.34 (ethyl acetate/dichloromethane, 70:30, v/v); UV (MeOH) λ_max_ nm (ε Lmol^−^^1^cm^−^^1^): 253 (57,447), 319 (13,993); IR (KBr) cm^−^^1^: 3,450–3,310 (NH_2_), 2,225 (CN), 1,677 (C=O), 1,643 (C=N); ^1^H-NMR: (DMSO-d_6_): δ = 2.41 (s, 3H, CH_3_), 6.02 (s, 2H, CH_2_), 7.30–7.98 (m, 15H, Ar-H + NH_2_), 8.65 (s, 1H, CH_ar-triazole_); ^13^C-NMR (DMSO-d_6_): δ = 21 (CH_3_), 52.1 (CH_2_), 72.1 (C-5), 116.7 (CN), 121.3 (CH_ar-triazole_), 125.3, 125.9, 127.8, 127.9, 128.3, 128.8, 129.3, 130.6, 130.7, 132.2, 132.4, 134.1, 135.3, 141, 146 (C_q-triazole_), 153.5 (C-2), 160 (C-4), 171.3 (C-6); MS-(+)ESI: *m/z* (%): 983 ([2M + H]^+^, 19), 514 ([M + Na]^+^, 7), 492 ([M+H]^+^, 100), MS-(-)ESI: *m/z* (%): 464 (26), 354 (8). 

*6-Amino-5-cyano-4-(4-methylphenyl)-1-(4-(1-(phenylthiomethyl)-1H-1,2,3-triazol-4-yl)phenyl)-2(1H)-pyrimidinone* (**4b**). White solid, yield (72%), C_27_H_21_N_7_OS, M = 491 gmol^−^^1^, mp 259–261 °C, R*_f_* = 0.27 (ethyl acetate/dichloromethane, 70:30, v/v); UV (MeOH) λ_max_ nm (ε Lmol^−^^1^cm^−^^1^): 251 (46,399), 318 (15,466); IR (KBr) cm^−^^1^: 3,450–3,310 (NH_2_), 2,226 (CN), 1,681 (C=O), 1,648 (C=N); ^1^H-NMR: (DMSO-d_6_): δ = 2.4 (s, 3H, CH_3_), 6.08 (s, 2H, CH_2_), 7.29–8.11 (m, 15H, Ar-H + NH_2_), 8.72 (s, 1H, CH_ar-triazole_); ^13^C-NMR (DMSO-d_6_): δ = 21 (CH_3_), 52.3 (CH_2_), 72.7 (C-5), 116.5 (CN), 121.7 (CH_ar-triazole_), 125.6, 127.4, 127.7, 128.6, 129.7, 130.5, 130.9, 132.1, 132.5, 133.9, 135.4, 140.9, 146.1 (C_q-triazole_), 154 (C-2), 160.1 (C-4), 171.4 (C-6); MS-(+)ESI: *m/z* (%): 983 ([2M + H]^+^, 20), 514 ([M + Na]^+^, 8), 492 ([M+H]^+^, 100), MS-(-)ESI: *m/z* (%): 464 (22), 354 (9). 

*6-Amino-4-benzyl-5-cyano-1-(3-(1-(phenylthiomethyl)-1H-1,2,3-triazol-4-yl)phenyl)-2(1H)-pyrimidin-one* (**4c**). White solid, yield (80%), C_27_H_21_N_7_OS, M = 491 gmol^−^^1^, mp 235–237 °C, R*_f_* = 0.36 (ethyl acetate/dichloromethane, 70:30, v/v); UV (MeOH) λ_max_ nm (ε Lmol^−^^1^cm^−^^1^): 250 (62,602), 318 (19,939); IR (KBr) cm^−^^1^: 3,450–3,310 (NH_2_), 2,208 (CN), 1,668 (C=O), 1,616 (C=N); ^1^H-NMR: (DMSO-d_6_): δ = 3.90 (s, 2H, CH_2_), 6.13 (s, 2H, CH_2_), 7.27–7.65 (m, 16H, Ar-H + NH_2_), 8.85 (s, 1H, CH_ar-triazole_); ^13^C-NMR (DMSO-d_6_): δ = 43.4 (CH_2_), 52.2 (CH_2_), 73.4 (C-5), 117 (CN), 122.3 (CH_ar-triazole_), 124.9, 125.6, 126.9, 127.7, 128.3, 129.2, 129.6, 131.2, 131.8, 133.1, 133.4, 134.7, 136.5, 140.9, 147.3 (C_q-triazole_), 154 (C-2), 159.6 (C-4), 175.7 (C-6); MS-(+)ESI: *m/z* (%): 983 ([2M + H]^+^, 19), 514 ([M + Na]^+^, 7), 492 ([M+H]^+^, 100), MS-(-)ESI: *m/z* (%): 464 (2), 354 (8). 

*6-Amino-4-benzyl-5-cyano-1-(4-(1-(phenylthiomethyl)-1H-1,2,3-triazol-4-yl)phenyl)-2(1H)-pyrimidin-one* (**4d**). White solid, yield (75%), C_27_H_21_N_7_OS, M = 491 gmol^−^^1^, mp 262–264 °C, R*_f_* = 0.30 (ethyl acetate/dichloromethane, 70:30, v/v); UV (MeOH) λ_max_ nm (ε Lmol^−^^1^cm^−^^1^): 248 (61,129), 319 (18,412); IR (KBr) cm^−^^1^: 3,450–3,310 (NH_2_), 2,226 (CN), 1,685 (C=O), 1,647 (C=N); ^1^H-NMR: (DMSO-d_6_): δ = 3.9 (s, 2H, CH_2_), 6.23 (s, 2H, CH_2_), 7.2–7.91 (m, 16H, Ar-H + NH_2_), 8.64 (s, 1H, CH_ar-triazole_); ^13^C-NMR (DMSO-d_6_): δ = 43.5 (CH_2_), 51.9 (CH_2_), 73.7 (C-5), 116.5 (CN), 120.5 (CH_ar-triazole_), 126, 126.9, 127.2, 129.1, 129.9, 129.9, 129.9, 131.9, 132.2, 134.2, 136.4, 141.2, 146.7 (C_q-triazole_), 153.5 (C-2), 159.3 (C-4), 175.6 (C-6); MS-(+)ESI: *m/z* (%): 983 ([2M + H]^+^, 22), 514 ([M + Na]^+^, 7), 492 ([M+H]^+^, 100), MS-(-)ESI: *m/z* (%): 464 (25), 354 (9). 

*6-Amino-5-cyano-1-(3-(1-(4-(isopropylamino)-6-(methylthio)-1,3,5-triazin-2-yl)-1H-1,2,3-triazol-4-yl)-phenyl)-4-(4-methylphenyl)-2(1H)-pyrimidinone* (**4e**). Pale yellow solid, yield (73%), C_27_H_25_N_11_OS, M = 551 gmol^−^^1^, mp 230–232 °C, R*_f_* = 0.39 (ethyl acetate/dichloromethane, 70:30, v/v); UV (MeOH) λ_max_ nm (ε Lmol^−^^1^cm^−^^1^): 248 (71,905), 318 (19,009); IR (KBr) cm^−^^1^: 3,450–3,310 (NH_2_ + NH), 2,208 (CN), 1,671 (C=O), 1,627 (C=N); ^1^H-NMR: (DMSO-d_6_): δ = 1.21 (d, 3H, *J* = 9 Hz, CH_3_), 1.23 (d, 3H, *J* = 9 Hz, CH_3_), 2.41 (s, 3H, CH_3_), 2.59 (s, 3H, -SCH_3_), 4.3 (m, 1H, CH), 7.25–7.77 (m, 11H, Ar-H + NH_2_ + NH), 8.1 (s, 1H, CH_ar-triazole_); ^13^C-NMR (DMSO-d_6_): δ = 21.2 (SCH_3_), 21.5 (CH_3_), 22.3 (CH_3_), 22.6 (CH_3_), 42.8 (CH), 73.8 (C-5), 117.2 (CN), 120.7 (CH_ar-triazole_), 126.3, 128.8, 129.3, 129.4, 131.3, 132.1, 132.1, 134.6, 135.9, 141.5, 146.4 (C_q-triazole_), 154 (C-2), 160.5 (C-4), 164, 171.8 (C-6), 182.1, 183.1; MS-(+)ESI: *m/z* (%): 574 ([M + Na]^+^, 7), 552 ([M+H]^+^, 100), MS-(-)ESI: *m/z* (%): 524 (64), 482 (7). 

*6-Amino-5-cyano-1-(4-(1-(4-(isopropylamino)-6-(methylthio)-1,3,5-triazin-2-yl)-1H-1,2,3-triazol-4-yl)-phenyl)-4-(4-methylphenyl)-2(1H)-pyrimidinone* (**4f**). white solid, yield (94%), C_27_H_25_N_11_OS, M = 551 gmol^−^^1^, mp 253–255 °C, R*_f_* = 0.31 (ethyl acetate/dichloromethane, 70:30, v/v); UV (MeOH) λ_max_ nm (ε Lmol^−^^1^cm^−^^1^): 251 (64,467), 319 (15,703); IR (KBr) cm^−^^1^: 3,450–3,310 (NH_2_ + NH), 2,225 (CN), 1,663 (C=O), 1,638 (C=N); ^1^H-NMR: (DMSO-d_6_): δ = 1.21 (d, 3H, *J* = 9 Hz, CH_3_), 1.23 (d, 3H, *J* = 9 Hz, CH_3_), 2.4 (s, 3H, CH_3_), 2.61 (s, 3H, -SCH_3_), 4.31 (m, 1H, CH), 7.21–7.75 (m, 11H, Ar-H + NH_2_ + NH), 8.21 (s, 1H, CH_ar-triazole_); ^13^C-NMR (DMSO-d_6_): δ = 21.1 (SCH_3_), 21.5 (CH_3_), 22.3 (CH_3_), 22.5 (CH_3_), 42.9 (CH), 73.8 (C-5), 116.9 (CN), 120.6 (CH_ar-triazole_), 124.2, 128.8, 129.6, 131.4, 133.3, 134.6, 136, 141.3, 146.7 (C_q-triazole_), 153.9 (C-2), 160.3 (C-4), 163.9, 171.7 (C-6), 182.1, 183.2; MS-(+)ESI: *m/z* (%): 574 ([M + Na]^+^, 7), 552 ([M+H]^+^, 100), MS-(-)ESI: *m/z* (%): 524 (54), 482 (9). 

*6-Amino-4-benzyl-5-cyano-1-(3-(1-(4-(isopropylamino)-6-(methylthio)-1,3,5-triazin-2-yl)-1H-1,2,3-triazol-4-yl)phenyl)-2(1H)-pyrimidinone* (**4g**). Yellowish solid, yield (88%), C_27_H_25_N_11_OS, M = 551 gmol^−^^1^, mp 241–243 °C, R*_f_* = 0.37 (ethyl acetate/dichloromethane, 70:30, v/v); UV (MeOH) λ_max_ nm (ε Lmol^−^^1^cm^−^^1^): 249 (50,416), 307 (27,274); IR (KBr) cm^−^^1^: 3,450–3,310 (NH_2_), 2,206 (CN), 1,670 (C=O), 1,629 (C=N); ^1^H-NMR: (DMSO-d_6_): δ = 1.21 (d, 3H, *J* = 9 Hz, CH_3_), 1.23 (d, 3H, *J* = 9 Hz, CH_3_), 2.59 (s, 3H, -SCH_3_), 3.9 (s, 2H, CH_2_), 4.3 (m, 1H, CH), 7.27–7.43 (m, 12H, Ar-H + NH_2_ + NH), 8.29 (s, 1H, CH_ar-triazole_); ^13^C-NMR (DMSO-d_6_): δ = 22.2 (SCH_3_), 22.3 (CH_3_), 22.5 (CH_3_), 42.8 (CH), 43.4 (CH_2_), 73.8 (C-5), 116.6 (CN), 120.8 (CH_ar-triazole_), 127.4, 128, 128.3, 129, 129.8, 130, 131.3, 135.2, 137.3, 146.5 (C_q-triazole_), 153.9 (C-2), 160.2 (C-4), 163.8, 164.2, 175.4 (C-6), 182.1, 183; MS-(+)ESI: *m/z* (%): 574 ([M + Na]^+^, 9), 552 ([M+H]^+^, 100), MS-(-)ESI: *m/z* (%): 524 (61), 482 (7). 

*6-Amino-4-benzyl-5-cyano-1-(4-(1-(4-(isopropylamino)-6-(methylthio)-1,3,5-triazin-2-yl)-1H-1,2,3-triazol-4-yl)phenyl)-2(1H)-pyrimidinone* (**4h**). White solid, yield (71%), C_27_H_25_N_11_OS, M = 551 gmol^−^^1^, mp 274–276 °C, R*_f_* = 0.32 (ethyl acetate/dichloromethane, 70:30, v/v); UV (MeOH) λ_max_ nm (ε Lmol^−^^1^cm^−^^1^): 250 (52,069), 308 (25,621); IR (KBr) cm^−^^1^: 3,450–3,310 (NH_2_), 2,226 (CN), 1,685 (C=O), 1,642 (C=N); ^1^H-NMR: (DMSO-d_6_): δ = 1.21 (d, 3H, *J* = 9 Hz, CH_3_), 1.23 (d, 3H, *J* = 9 Hz, CH_3_), 2.60 (s, 3H, -SCH_3_), 3.9 (s, 2H, CH_2_), 4.3 (m, 1H, CH), 7.36–7.47 (m, 12H, Ar-H + NH_2_ + NH), 8.3 (s, 1H, CH_ar-triazole_); ^13^C-NMR (DMSO-d_6_): δ = 21.5 (SCH_3_), 22.3 (CH_3_), 22.5 (CH_3_), 42.8 (CH), 43.4 (CH_2_), 73.8 (C-5), 116.5 (CN), 120.7 (CH_ar-triazole_), 127.2, 127.8, 128.9, 129.6, 131.1, 135, 137.1, 146.5 (C_q-triazole_), 154.1 (C-2), 159.7 (C-4), 163.6, 164, 175.4 (C-6), 182.1, 183.1; MS-(+)ESI: *m/z* (%): 574 ([M + Na]^+^, 7), 552 ([M+H]^+^, 100), MS-(-)ESI: *m/z* (%): 524 (56), 482 (7). 

*(Z)-Ethyl-2-(4-(4-(3-(6-amino-5-cyano-4-(4-methylphenyl)-2-oxopyrimidin-1(2H)-yl)phenyl)-1H-1,2,3-triazol-1-yl)benzylidene)-7-methyl-3-oxo-5-phenyl-3,5-dihydro-2H-thiazolo[3,2-a]pyrimidine-6-carboxylate* (**4i**). Golden yellow solid, yield (84%), C_43_H_33_N_9_O_4_S, M = 771 gmol^−^^1^, mp 285–287 °C, R*_f_* = 0.28 (ethyl acetate/dichloromethane, 50:50, v/v); UV (MeOH) λ_max_ nm (ε Lmol^−^^1^cm^−^^1^): 248 (95,989), 308 (35,851); IR (KBr) cm^−^^1^: 3,450–3,310 (NH_2_), 2,211 (CN), 1,715 (C=O, ester), 1,677 (C=O); ^1^H-NMR: (DMSO-d_6_): δ = 1.13 (t, 3H, *J* = 6 Hz, CH_3_), 2.40 (s, 3H, CH_3_), 2.41 (s, 3H, CH_3_), 4.04 (q, 2H, *J* = 6 Hz, CH_2_), 6.06 (s, 1H, C-CH-N), 7.31–8.14 (m, 20H, Ar-H + NH_2_), 9.47 (s, 1H, CH_ar-triazole_); ^13^C-NMR (DMSO-d_6_): δ = 14.3 (CH_3_-CH_2_), 21.5 (CH_3_), 22.9 (CH_3_), 55.5 (C-CH-N), 60.7 (CH_3_-CH_2_), 72.6 (C-5), 109.4, 117.1 (CN), 120.4, 120.8 (CH_ar-triazole_), 121.3, 125.9, 126.7, 127.9, 128.8, 129.1, 129.2, 129.3, 131.5, 131.9, 132.1, 132.5, 133.4, 134.6, 135.9, 137.7, 140.7, 141.5, 147.3 (C_q-triazole_), 151.5, 154 (C-2), 155.7, 160.5 (C-4), 164.6 (C=O), 165.3, 171.9 (C-6); MS-(+)ESI: *m/z* (%): 794 ([M + Na]^+^, 3), 772 ([M+H]^+^, 100), MS-(-)ESI: *m/z* (%): 744 (10). 

*(Z)-Ethyl-2-(4-(4-(4-(6-amino-5-cyano-4-(4-methylphenyl)-2-oxopyrimidin-1(2H)-yl)phenyl)-1H-1,2,3-triazol-1-yl)benzylidene)-7-methyl-3-oxo-5-phenyl-3,5-dihydro-2H-thiazolo[3,2-a]pyrimidine-6-carboxylate* (**4j**). Golden yellow solid, yield (72%), C_43_H_33_N_9_O_4_S, M = 771 gmol^−^^1^, mp 292–294 °C, R*_f_* = 0.25 (ethyl acetate/dichloromethane, 50:50, v/v); UV (MeOH) λ_max_ nm (ε Lmol^−^^1^cm^−^^1^): 249 (84,424), 307 (38,164); IR (KBr) cm^−^^1^: 3,450–3,310 (NH_2_), 2,225 (CN), 1,727 (C=O, ester), 1,685 (C=O); ^1^H-NMR: (DMSO-d_6_): δ = 1.15 (t, 3H, *J* = 6 Hz, CH_3_), 2.41 (s, 3H, CH_3_), 2.43 (s, 3H, CH_3_), 4.11 (q, 2H, *J* = 6 Hz, CH_2_), 6.02 (s, 1H, C-CH-N), 7.29–8.20 (m, 20H, Ar-H + NH_2_), 9.32 (s, 1H, CH_ar-triazole_); ^13^C-NMR (DMSO-d_6_): δ = 14.3 (CH_3_-CH_2_), 21.5 (CH_3_), 22.9 (CH_3_), 55.5 (C-CH-N), 60.6 (CH_3_-CH_2_), 72.6 (C-5), 109.4, 117.4 (CN), 120.7, 120.8 (CH_ar-triazole_), 122, 126.2, 126.7, 128.1, 129, 129.3, 131.6, 132, 132.2, 132.6, 133.6, 134.5, 136.1, 137.7, 140.6, 141.4, 147.5 (C_q-triazole_), 151.7, 152.2 (C-2), 156.2, 161.2 (C-4), 165.1 (C=O), 165.6, 172.5 (C-6); MS-(+)ESI: *m/z* (%): 794 ([M + Na]^+^, 3), 772 ([M+H]^+^, 100), MS-(-)ESI: *m/z* (%): 744 (7). 

*(Z)-Ethyl-2-(4-(4-(3-(6-amino-4-benzyl-5-cyano-2-oxopyrimidin-1(2H)-yl)phenyl)-1H-1,2,3-triazol-1-yl)benzylidene)-7-methyl-3-oxo-5-phenyl-3,5-dihydro-2H-thiazolo[3,2-a]pyrimidine-6-carboxylate* (**4k**). Golden yellow solid, yield (81%), C_43_H_33_N_9_O_4_S, M = 771 gmol^−1^, mp 277–279 °C, R*_f_* = 0.32 (ethyl acetate/dichloromethane, 50:50, v/v); UV (MeOH) λ_max_ nm (ε Lmol^−1^cm^−1^): 250 (72,859), 318 (27,756); IR (KBr) cm^−1^: 3,450–3,310 (NH_2_), 2,212 (CN), 1,721 (C=O, ester), 1,671 (C=O); ^1^H-NMR: (DMSO-d_6_): δ = 1.12 (t, 3H, *J* = 6 Hz, CH_3_), 2.40 (s, 3H, CH_3_), 3.9 (s, 2H, CH_2_), 4.09 (q, 2H, *J* = 6 Hz, CH_2_), 5.98 (s, 1H, C-CH-N), 7.33–8.02 (m, 21H, Ar-H + NH_2_), 9.33 (s, 1H, CH_ar-triazole_); ^13^C-NMR (DMSO-d_6_): δ = 14.3 (CH_3_-CH_2_), 22.9 (CH_3_), 43.5 (CH_2_), 55.4 (C-CH-N), 60.7 (CH_3_-CH_2_), 73.4 (C-5), 110.2, 116.5 (CN), 119.9, 121.2 (CH_ar-triazole_), 122, 126, 126.6, 128.3, 128.9, 129, 129.8, 130.1, 131.5, 132, 132.4, 132.9, 134.2, 134.7, 136.1, 137.5, 139.9, 142, 146.7 (C_q-triazole_), 150.9, 153.6 (C-2), 156.2, 159.6 (C-4), 164.5 (C=O), 166.2, 175.8 (C-6); MS-(+)ESI: *m/z* (%): 794 ([M + Na]^+^, 3), 772 ([M+H]^+^, 100), MS-(-)ESI: *m/z* (%): 744 (9).

*(Z)-ethyl-2-(4-(4-(4-(6-amino-4-benzyl-5-cyano-2-oxopyrimidin-1(2H)-yl)phenyl)-1H-1,2,3-triazol-1-yl)benzylidene)-7-methyl-3-oxo-5-phenyl-3,5-dihydro-2H-thiazolo[3,2-a]pyrimidine-6-carboxylate* (**4l**). Golden yellow solid, yield (68%), C_43_H_33_N_9_O_4_S, M = 771 gmol^−1^, mp 281–283 °C, R*_f_* = 0.61 (ethyl acetate/dichloromethane, 50:50, v/v); UV (MeOH) λ_max_ nm (ε Lmol^−1^cm^−1^): 253 (90,207), 319 (15,381); IR (KBr) cm^−1^: 3,450–3,310 (NH_2_), 2,226 (CN), 1,728 (C=O, ester), 1,676 (C=O); ^1^H-NMR: (DMSO-d_6_): δ = 1.15 (t, 3H, *J* = 6 Hz, CH_3_), 2.41 (s, 3H, CH_3_), 3.89 (s, 2H, CH_2_), 4.12 (q, 2H, *J* = 6 Hz, CH_2_), 6.00 (s, 1H, C-CH-N), 7.28–8.46 (m, 21H, Ar-H + NH_2_), 9.40 (s, 1H, CH_ar-triazole_); ^13^C-NMR (DMSO-d_6_): δ = 14.3 (CH_3_-CH_2_), 22.9 (CH_3_), 43.5 (CH_2_), 56.2 (C-CH-N), 60.7 (CH_3_-CH_2_), 73.8 (C-5), 112.5, 117.2 (CN), 120.3, 120.9 (CH_ar-triazole_), 121.9, 126.3, 128.6, 129.4, 129.5, 129.9, 131.6, 131.8, 133.5, 133, 135.2, 135.4, 135.9, 137.6, 140.1, 142.2, 147.7 (C_q-triazole_), 149.5, 153.9 (C-2), 157, 159.9 (C-4), 164.6 (C=O), 167.2, 174.9 (C-6); MS-(+)ESI: *m/z* (%): 794 ([M + Na]^+^, 3), 772 ([M+H]^+^, 100), MS-(-)ESI: *m/z* (%): 744 (6).
